# Effects of Multidisciplinary Rehabilitation on Chronic Fatigue in Multiple Sclerosis: A Randomized Controlled Trial

**DOI:** 10.1371/journal.pone.0107710

**Published:** 2014-09-18

**Authors:** Marc B. Rietberg, Erwin E. H. van Wegen, Isaline C. J. M. Eyssen, Gert Kwakkel, the MS study group

**Affiliations:** Department of Rehabilitation Medicine, MOVE Research Institute Amsterdam, VU University Medical Center, Amsterdam, The Netherlands; University Medical Center Göttingen, Germany

## Abstract

**Background:**

Several rehabilitation programmes aim at reducing the impact of fatigue in MS patients. Acute and chronic fatigue should require different management.

**Objectives:**

To assess the effects of individually tailored, multidisciplinary outpatient rehabilitation (MDR) on chronic fatigue.

**Methods:**

Forty-eight ambulatory MS patients with chronic fatigue were randomized to MDR or to MS–nurse consultation. Fatigue was assessed by the Checklist Individual Strength (CIS-20R). Secondary outcomes included the Modified Fatigue Impact Scale, Fatigue Severity Scale, Functional Independence Measure, Disability and Impact Profile (DIP), Multiple Sclerosis Impact Scale and the Impact on Participation and Autonomy (IPA).

**Results:**

The primary outcome measure CIS-20R overall score showed no significant differences between groups at 12 weeks (P = 0.39) and 24 weeks follow-up (P = 0.14), nor for subscales (t = 12 and t = 24, 0.19≤P≤0.88). No significant within-group effects were found for both groups with respect to the primary (0.57≤p≤0.97) and secondary (0.11≤p≤0.92) outcome measures from baseline to 12 or 24 weeks.

**Conclusion:**

Multidisciplinary rehabilitation was not more effective in terms of reducing self-reported fatigue in MS patients compared to MS-nurse consultation. Our results suggest that chronic fatigue in patients with MS may be highly invariant over time, irrespective of interventions.

**Trial Registration:**

controlled-trials.com ISRCTN05017507

## Introduction

Fatigue is a common and disabling symptom in people with multiple sclerosis (MS), and is considered to be one of the main causes of impaired daily activities and reduced quality of life [1]. Several pathophysiological mechanisms for fatigue, such as dysregulation of the immune system, impaired nerve conduction, and neuro-endocrine and neurotransmitter changes [1]–[3] have been suggested to explain fatigue in MS; however, the exact mechanism is still not well known. Despite, the poor understanding of the etiology, it is well accepted that fatigue is subjective and multidimensional in nature [4], [5]. Based on assumed underlying pathophysiological mechanisms, the construct of fatigue in MS is often classified either into central and peripheral [1] or into primary and secondary fatigue [2]. Although fatigue is considered to be a direct consequence of pathophysiological mechanisms of the MS disease process [1], factors secondary to MS, such as pain and muscle spasms, and concomitant conditions such as viral infections, urinary infections, pregnancy, alcohol or substance abuse and depression may contribute to feelings of fatigue [1], [2], [6], [7]. The Multiple Sclerosis Council for Clinical Practice Guidelines (MSCCPG) [6] distinguishes chronic persistent fatigue from acute fatigue. Chronic persistent fatigue is defined as “being present for any amount of time on 50 percent of the days, for more than 6 weeks”, whereas acute fatigue is defined as “new or significant increase in feelings of fatigue in the previous six weeks”. Both chronic and acute fatigue limit functional activities and quality of life, but may require different management strategies [6].

The pathophysiological basis of fatigue remains unclear and consequently effective treatment is limited. A number of clinical trials have tested a variety of pharmacological and non-pharmacological interventions for fatigue in MS. Several drugs such as amantadine, pemoline, modafinil, and aminopyridine have been advocated with respect to their effect on fatigue in MS. The evidence for effects of pharmacological treatment is not established, with exception of amantadine, which might be of benefit to some MS patients [8]–[13]. Several non-pharmacological treatment components, such as aerobic training [14]–[17], cognitive behaviour therapy [12] and energy management strategies [18], [19] aim at reducing the impact of fatigue on patients with MS. In view of the multidimensional character of fatigue in MS it seems obvious to manage this problem with a tailored, multidisciplinary approach [3], [7], [20]. However, only a few randomized clinical trials have evaluated the effect of a combination of these treatment components on *chronic* fatigue in MS as the main focus of intervention, using fatigue as the primary measure of outcome [21]. As a consequence, the optimal management strategy for treatment remains elusive [20].

According to the MSCCPG [6], chronic and acute fatigue require different management approaches. However, most studies performed on treatment of fatigue have used mixed samples without descriptive modifiers to discriminate between acute, intermitted fatigue and chronic persistent fatigue. The present study specifically focussed on optimising self management behaviour with regards to chronic fatigue as this highly impacts long term daily functioning and quality of life.

Therefore, the objective of the present study was to investigate the effects of an individually tailored, multidisciplinary outpatient rehabilitation programme as compared to monodisciplinary consultation by an MS nurse on chronic fatigue in MS.

## Methods

### Ethics statement

The RCT was approved by the medical ethics committee of the VU University Medical Center, Amsterdam, Registration number 2005/72.

### Supporting information

The protocol for this trial and supporting CONSORT checklist are available as supporting information; see Checklist S1 and Protocol S1.

### Trial design

A single-blinded, randomized controlled trial (RCT) was conducted, with double baseline assessment at week -1 and week 0, followed by one post-intervention assessment at 12 weeks and a follow-up assessment at 24 weeks. In order- of admission, MS patients with chronic fatigue were randomly assigned to a multidisciplinary outpatient rehabilitation programme (MDR) or to outpatient MS-nurse consultation (NC). The randomization procedure was concealed and based on computer-generated block randomization with block sizes of 8. No stratification was applied. Implementation of the random allocation was done by means of sequentially numbered sealed opaque envelops. All patients gave written informed consent. The trial protocol has been registered after enrolment of participants, because trial registration was not required by the ethics committee at the time the trial started.

### Participants

Patients were included from the outpatient clinic of the university medical center, if they were (1) older than 18 years; (2) diagnosed with MS according to the McDonald criteria [22]; (3) suffering from chronic fatigue according to the MSCCPG definition [6]; and (4) able to walk.

Patients were excluded in case of (1) current MS relapse, (2) pregnancy, (3) current infection (cystitis), (4) alcohol or substance abuse, (5) physical conditions like muscle spasm or pain contributing to sleep problems, (6) pharmacological treatment for fatigue that was started in the past 3 months, or (7) depressive symptomatology importantly contributing to fatigue according to the Hospital Anxiety and Depression Scale (HADS) [23]. A score of 8 or higher on the depression scale was classified as depression [24].

### Procedure

Eligible patients were screened for the inclusion and exclusion criteria by a neurologist. Due to slow recruitment we were not able to keep our original time frame for inclusion of patients between 2005 and 2008. Recruitment started in January 2006 and the last follow-up assessment was performed in December 2009. Before patients were allocated to a treatment group, the neurologist completed a standardized fatigue screening questionnaire. It is a structured approach which starts with identification of the most important daily problems related to fatigue as perceived by the patient, such as dividing time between rest and activity, improving or maintaining physical condition and coping with MS symptoms. Moreover, patients were asked to indicate their preferences regarding the sequence in treatment for their individual identified problems. Subsequently, a multidisciplinary team, consisting of a neurologist, rehabilitation doctor, occupational therapist, physiotherapist, social worker, MS nurse and medical psychologist, discussed the results of the fatigue screening by the neurologist and a tailored pathway of referral was determined for each individual patient. Then, patients were randomly allocated to MDR or to NC. Patients to MDR were referred to one or more disciplines that were professionally linked to the fatigue management problems of interest to each patient.

A trained independent observer who was blinded to treatment allocation collected all demographic information, disease characteristics and outcome data, in individual meetings at the patient’s own home. Information concerning treatment content and contact time for both groups was kept in a patient diary by the therapist. Changes in medication intake were recorded by the therapist. The participants and the observer were instructed not to discuss the treatment procedure. At the end of the trial the independent observer was asked to guess the intervention allocated to each participant.

### Interventions

#### Multidisciplinary Rehabilitation programme (MDR)

Patients assigned to MDR received an individually tailored programme that focussed on optimising self management behaviour in daily life activities on the domains of physical fitness, behaviours or cognitions that perpetuate fatigue, and energy conservation. For addressing this therapy goals participants received physical therapy (PT), or occupational therapy (OT), or social work (SW), or any combination of these treatments. For PT, the number of treatment sessions was predefined, whereas for the other intervention types, the number of sessions was on an as-needed basis, with a minimum of 2 sessions. In addition to the outpatient treatment sessions, the MS patients were given homework assignments. The participating disciplines treated MS-related fatigue according to specific treatment programmes, as described below.

### Physical therapy (PT)

An individualized exercise training program was devised to address the ‘reconditioning’ factor, aimed at improving physical fitness. The 12-week training programme consisted of two 45-minute sessions a week of supervised aerobic training in circuit style, performed individually or in classes. Maximal aerobic capacity of each participant was estimated by means of a submaximal bicycle ergometer test. Moderate intensity was defined as 50–70% VO2-peak steady-state endurance training [25], [26]. Various fitness devices (e.g. bicycle ergometer, rowing ergometer, stair walker) were used in blocks of six minutes, in order to offer a total body work-out.

### Occupational therapy (OT)

Patients were referred to occupational therapy to address the factors of ‘dividing time between rest and activity’, ‘work, education, leisure time and social contacts’, ‘sitting and walking’ and ‘personal care’. During a one-hour session, intervention goals were set, which were evaluated in follow-up consultations. Fatigue management skills were taught to help with the application of coping strategies, energy conservation [18], time management, efficient body mechanics and task performance [27].

### Social work (SW)

Patients were referred to social work to address the factors of ‘support from the environment’, ‘conflicts at work or with social services’, and ‘coping with MS’. The social worker provided psychosocial support through counselling and practical assistance. Goals were set during a one-hour session, and subsequently evaluated in follow up consultations. The psychosocial support, used the techniques of skilled listening, encouragement to ventilate feelings, normalization of feelings and advice regarding coping strategies, coupled with practical help to enable both patient and family to cope with difficult circumstances identified.

#### MS-Nurse consultation (NC)

Patients allocated to the NC group received consultation according to the Nursing Intervention Classification [28]. Goals were set during a one-hour session, and subsequently evaluated in follow-up consultations every three weeks. The nurse discussed general principles of planning of activities, priority setting, energy conservation, accepting help from others with daily life activities or use of devices. Physical activity was recommended. Patients were advised on nutrition and alcohol and drug intake. In addition to the consultation sessions, the patients were given homework assignments.

### Outcomes

#### Primary outcome measure

Fatigue was assessed with the Checklist Individual Strength (CIS-20R). The CIS-20R [29] assesses fatigue during the past 2 weeks and consists of four domains: subjective experience of fatigue; reduced motivation; reduced activity and reduced concentration. The CIS-20R consists of twenty statements for which the participant has to indicate on a seven-point scale to what extent the particular statement applies to him or her, ranging from ‘Yes, that is true’ to ‘No, that is not true’. An overall score and sub-scores for the domains are calculated. The instrument has been validated for MS patients [30] and shows good test-retest reliability.

#### Secondary outcome measures

Secondary outcomes included two other self-report questionnaires for fatigue viz. the Modified Fatigue Impact Scale (MFIS) [31], [32] and the Fatigue Severity Scale (FSS) [33], as well as the Functional Independence Measure (FIM) [34], the Disability and Impact Profile (DIP) [35], [36], the Multiple Sclerosis Impact Scale (MSIS-29) [37] and the Impact on Participation and Autonomy (IPA) instrument [38]. In the original trial protocol we included the short-form health survey (SF-36) to measure health-related quality of life. The SF-36 was replaced by a disease specific outcome measure of the impact of multiple sclerosis, the MSIS-29 [37].

### Statistical analysis

To control for learning effects by repeated assessments, the second baseline assessment at week 0 was used for analysis. Demographic variables, disease characteristics and primary and secondary outcomes at baseline were summarized using descriptive statistics. Baseline differences between MDR and NC were checked by using the independent samples t-test for continuous variables viz. age and number of years since diagnosis. Normality was checked based on visual plots. The chi-square test was used for the categorical variables MS type and gender, whereas the Mann-Whitney U test was applied for outcomes at ordinal scales.

Treatment effects were tested by calculating changes scores from baseline to 12 weeks and from 12 to 24 weeks. Subsequently, the Mann-Whitney U test was used to test differences in change scores between the MDR group and the NC group at 12 and 24 weeks. All analyses used a two-tailed significance level of 0.05. For conducting these statistical analyses SPSS, version 20.0 was used.

We expected a significant reduction of 14 points on the CIS-20R total score in favor of the MDR group. Sample size calculation using Gpower software (Kiel University, Germany) showed that minimally 24 patients were required per arm of the trial, assuming a drop-out of 10%, a statistical power of 80% (to prevent type II error) to reject the H0 hypothesis and two-tailed alpha of 0.05 (CIS-20R mean 85, sd 16) [39].

## Results

**Figure 1:**
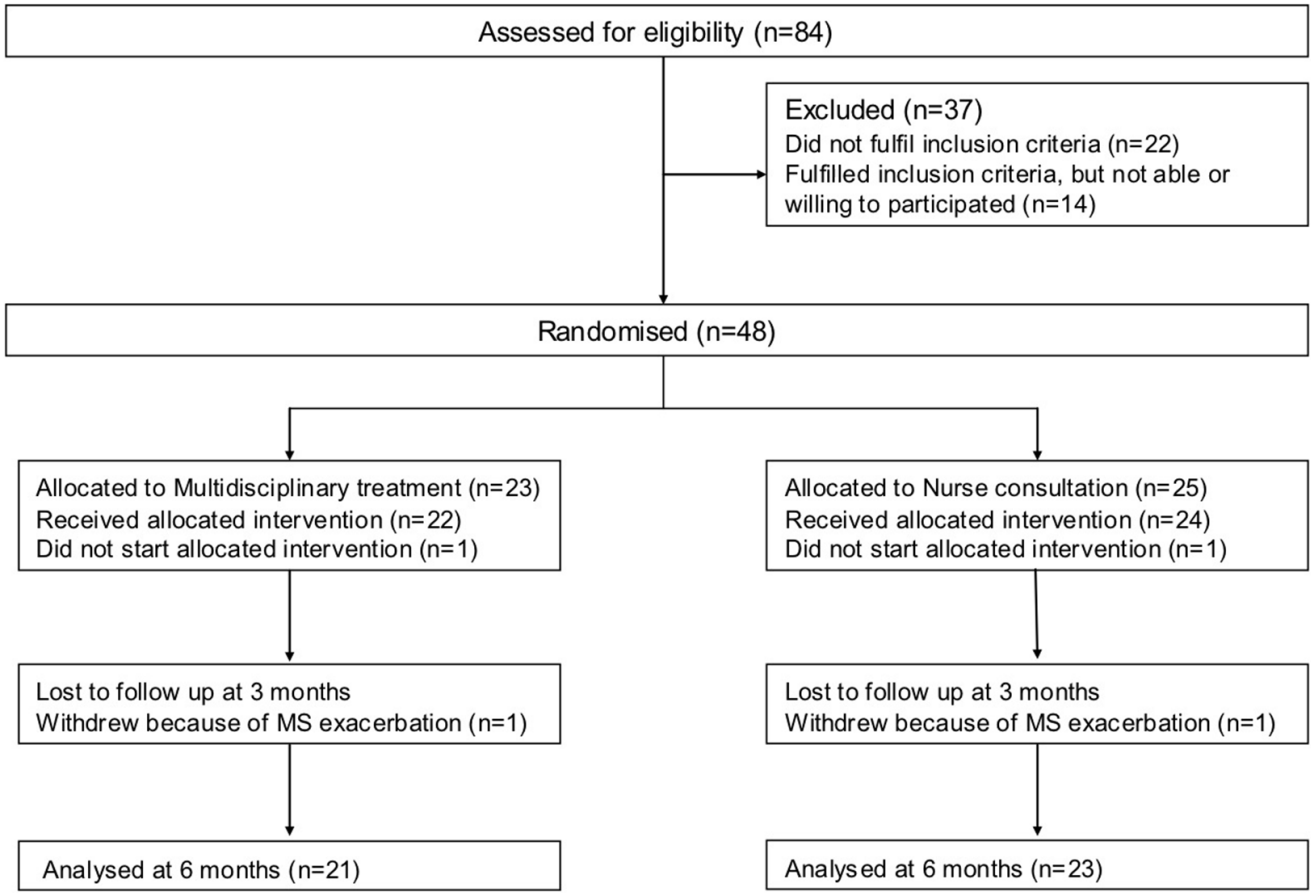
Flow of patients through an RCT comparing Multidisciplinary Rehabilitation with Nurse consultation.

Figure 1 shows the flowchart of the trial. Eighty-four patients with MS were assessed for eligibility. Forty-eight patients suffering from chronic fatigue were randomly assigned to MDR (N = 23) or NC (N = 25). Of the remaining patients, 22 did not meet the inclusion criteria (26.2%) and 14 patients (16.7%) were unable or unwilling to participate.

The number of drop-outs and reasons for dropping out were equally divided over the MDR and NC groups. Reasons for drop-out were withdrawal from participation because of lack of motivation (N = 2) and exacerbation of MS (N = 2).

No statistically significant baseline differences were found (Table 1). The results of the referral pathway with priority setting for the fatigue management factors of interest to the patients were comparable for the MDR and NC groups. Both groups gave priority to optimising the division of time between rest and activity, as well as to work, education, leisure and social contacts. Furthermore, both groups gave priority to ‘optimise coping with MS’. The only difference between the groups was that the multidisciplinary rehabilitation group also gave priority to ‘optimising physical condition’.

**Table 1:** Baseline characteristics of people with MS allocated to MDR or NC.

	Multidisciplinary rehabilitation	Nurse consultation	P value
Patients’ characteristics	(N = 23)	(N = 25)	
	Mean (SD)	Mean (SD)	
No (%) of women	14 (60.9)	17 (68)	0.53
Age (years)	45 (9.9)	47 (8.6)	0.38
No (%) type MS; PP/SP/RR	2 (8.7)/5 (21.7)/16 (69.6)	6 (24)/7 (28)/12 (48)	0.15
Years since diagnosis	7 (6.6)	8 (6.1)	0.62
	**Median (IQR)**	**Median (IQR)**	
EDSS	3 (3)	4 (2)	0.18
HADS Anxiety (0–21)	3 (4)	3 (4)	0.62
HADS Depression (0–21)	2 (1)	2 (2)	0.69
**Primary outcome**			
CIS-20R (20–140)	78 (13.5)	79 (13)	0.90
Subjective feeling (8–56)	30 (8)	31 (7)	0.65
Concentration (5–35)	21 (3.5)	20 (6)	0.45
Motivation (4–28)	15 (5)	16 (5)	0.82
Physical activity (3–21)	11 (4)	12 (4)	0.85
**Secondary outcome**			
FSS (9–63)	52 (11.5)	48 (12)	0.59
MFIS (0–84)	43 (18.75)	36 (14.5)	0.45
Physical subscale (0–36)	22.5 (9.25)	18 (7)	0.17
Cognitive subscale (0–40)	16.5 (14)	15 (8)	0.92
Psychosocial subscale (0–8)	3.5 (2)	3 (1.5)	0.84
FIM (18–126)	118.5 (6.75)	122 (11)	0.31
MSIS Physical (0–100)	53 (20)	43 (19)	0.41
MSIS Psychological (0–100)	18 (6.5)	17 (6)	0.49
DIP Symptoms	0.33 (0.33)	0 (0.33)	0.14
DIP Mobility	0.1 (0.1)	0 (0.10)	0.79
DIP Self-care	0 (0)	0 (0)	0.88
DIP Social Activities	0 (0)	0 (0.10)	0.45
DIP Communication	0 (0)	0 (0)	0.34
DIP Psychological status	0 (0)	0 (0)	0.61
IPA subscales			
Autonomy indoors	0.43 (0.89)	1 (0.93)	0.12
Family role	1.71 (1.14)	1.43 (1.07)	0.77
Autonomy outdoors	1.60 (0.80)	1.6 (0.80)	0.54
Social life and relationships	1.14 (0.75)	1.14 (0.64)	0.79
Work and education	2.2 (1.5)	1.74 (0.83)	0.29
IPA problem experience			
Mobility	1 (1)	1 (1)	0.51
Self care	1 (2)	1 (2)	0.83
Activities around the house	1 (0)	1 (0)	0.40
Looking after money	0 (1)	1 (1)	0.23
Leisure	1 (0.5)	1 (0)	0.40
Social life and relationships	1 (1)	1 (1)	0.91
Helping, supporting other people	1 (1)	1 (0)	0.39
Paid or voluntary work	1 (1)	1 (1)	0.76
Education and training	0 (1)	1 (1)	0.75

PP = Primary Progressive; SP = Secondary Progressive; RR = Relapsing Remitting; IQR = interquartile range; EDSS = Expanded Disability Status Scale; CIS-20R = Checklist Individual Strength; FSS = Fatigue Severity Scale; MFIS = Modified Fatigue Impact Scale, MSIS = Multiple Sclerosis Impact Scale; FIM = functional independence measure; DIP = Disability and Impact Profile (DIP), IPA = Impact on Participation and Autonomy.

The average total treatment time per participant was 280 minutes (SD 187 min) for multidisciplinary rehabilitation and 163 minutes (SD 106 min) for nurse consultation. Sixty-seven percent of the patients in the MDR group were given homework assignments at 26 percent of the visits at the outpatient clinic, while the corresponding numbers for the NC group were 87% and 68%, respectively. The adherence rate for doing the homework was 96% in the MDR group and 89% in the NC group.

In the MDR group, only one patient was given homework by the PT, which included walking outside at a rapid pace. Homework set by the OT involved filling out an ‘activity list for fatigue’ or making a ‘weekly schedule’ to manage fatigue through efficient performance and better planning. The homework given by SW mostly involved asking patients to express their view on fatigue and record negative thoughts or thoughts about fatigue. The NC group had a broader spectrum regarding the type and content of homework given, mainly aimed at ‘planning the day - saving energy’, ‘getting help’ and ‘engaging in sports’.

Medications were changed for 39% of the participants of the NC group and 19% of those in the MDR group. In both arms of the trial, participants used urological medication (anticholinergics oxybutynin and tolterodine), Viagra (sildenafil citrate) and anti-allergy drugs (cetirizine, chromoglicine acid).

The observer correctly guessed 24 out of 44 allocations, resulting in a Cohen’s kappa of 0.09, suggesting that the procedures for keeping the observers blinded for treatment allocation were sufficient.

### Outcomes

**Figure 2:**
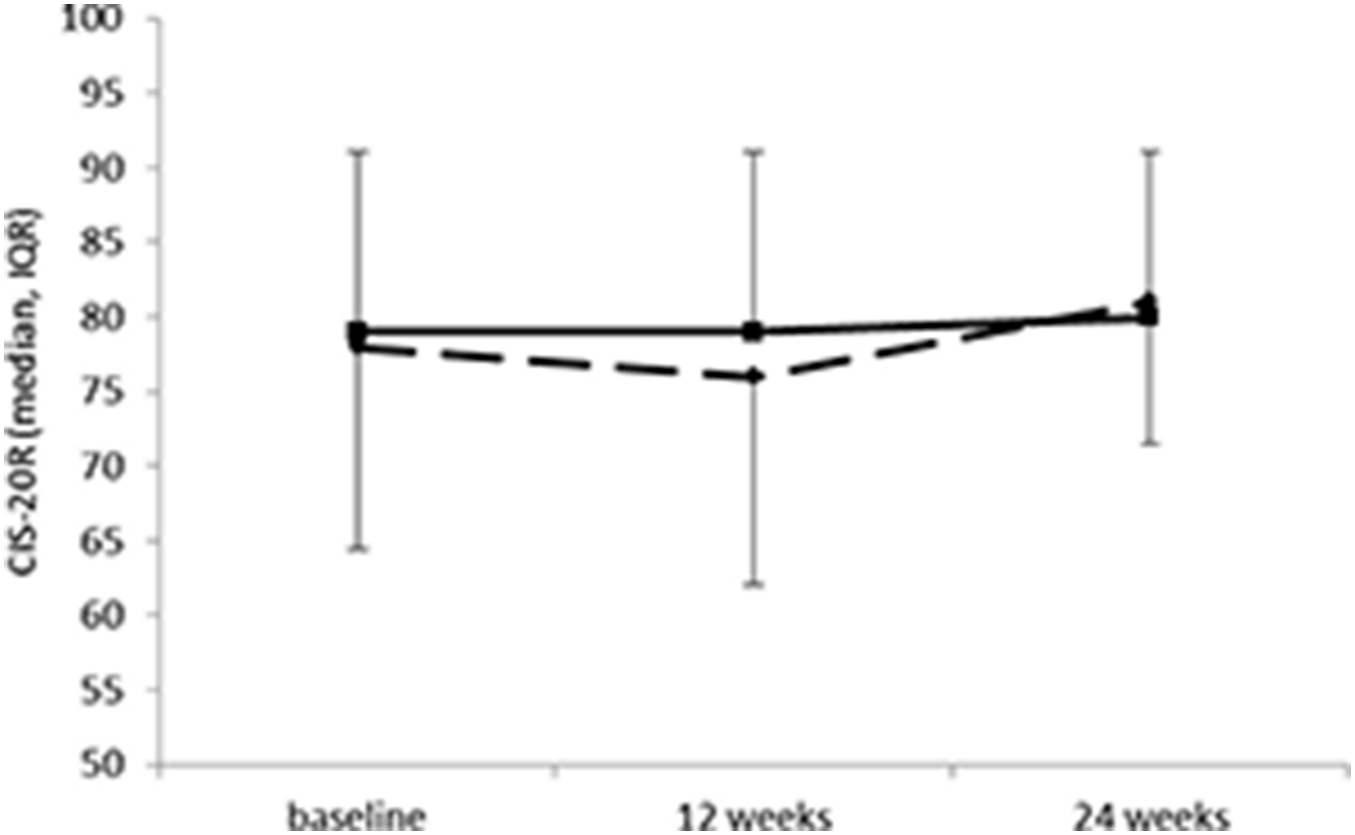
Median scores and inter quartile ranges on the CIS-20R of the Multidisciplinary Rehabilitation group, N = 21 (dotted line) and the MS-Nurse consultation group, N = 23 (solid line).

Upon visual plot, most outcomes, including the CIS-20R and subscales were non-normally distributed. Figure 2 presents the median scores and inter quartile ranges (IQR) for the CIS-20R at baseline, 12 and 24 weeks for both groups. Table 2 shows the median scores at 12 and 24 weeks, the mean changes scores from baseline to 12 weeks and from 12 to 24 weeks, and the effects between groups for fatigue outcomes. The primary measure of outcome, the total CIS-20R score, showed no statistically significant differences between MDR and NC at the post-intervention assessment (P = 0.39), nor at the follow-up assessment (P = 0.14). No significant group differences were found for the subscales of the CIS-20R (0.19≤P≤0.88) for either of the assessment moments. No significant group differences were found for the other secondary measures of outcome with respect to fatigue (FSS, and MFIS and subscales).

**Table 2:** Fatigue outcomes at 12 and 24 weeks for people with MS randomized to multidisciplinary rehabilitation or Nurse consultation.

	MDR		NC		MDR		NC		Between-group differences	
	12 weeks(N = 21)	24 weeks(N = 21)	12 weeks(N = 23)	24 weeks(N = 23)	0–12 weeksΔ score	12–24 weeksΔ score	0–12 weeksΔ score	12–24 weeksΔ score	0–12 weeks Meandiff.; P	12–24 weeks Meandiff.; P
**Primary outcome**										
CIS-20R	76 (14)	81 (9.5)	79 (12)	80 (11)	−0.8 (7.1)	3,4 (8,8)	2.2 (10.3)	−1.0 (8.8)	3.0; 0.39	4.4; 0.14
Subjective feeling	31 (8.5)	35 (8)	33 (5)	32 (5)	0.6 (3.2)	2,1 (5,1)	1.7 (5.0)	−0.6 (6.1)	1.1; 0.55	2.7; 0.28
Concentration	18 (5)	21 (3)	19 (3)	20 (3)	−1.1 (3.8)	1,3 (3,7)	−0.3 (3.3)	−0.2 (3.0)	0.8; 0.40	1.1; 0.19
Motivation	15 (4)	16 (5)	14 (5)	14 (10)	−0.6 (3.1)	0,1 (3,3)	0.3 (3.3)	0.0 (2.8)	0.9; 0.27	0.1; 0.71
Physical activity	11 (4)	12 (3.5)	13 (3)	13 (3)	0,3 (2,1)	0,3 (2,5)	0.6 (2.9)	−0.3 (2.1)	0.9; 0.88	0.6; 0.64
**Secondary outcomes**										
FSS	49 (7.5)	51 (9.5)	50 (13)	47 (11)	−1.6 (7.1)	0,5 (7,9)	0.3 (8.5)	−1.3 (7.8)	1.9; 0.47	1.8; 0.27
MFIS	42 (24)	42 (11)	37 (20)	42 (21)	−1,2 (9,5)	1,9 (11,2)	−0.6 (13.8)	3.9 (11.9)	0.6; 0.71	2,0; 0.78
Physical	21 (9.5)	20 (7.5)	17 (8)	21 (9)	−1,1 (4,4)	1.0 (4.6)	−0.6 (6.3)	2.2 (5.7)	0.5; 0.77	1.2; 0.53
Cognitive	16 (12.5)	16 (5.5)	14 (11)	17 (17)	−0,1 (6,3)	0,6 (7.7)	0.1 (7.4)	1.4 (9.7)	0.2; 0.67	0.8; 0.99
Psychosocial	4 (2)	4 (2)	4 (3)	4 (2)	0,1 (1,5)	0.3 (1,6)	−0.1 (1.9)	0.3 (1.6)	0.2; 0.90	0; 0.71

Values are medians (inter quartile ranges). Change scores are means (standard deviations). MDR = multidisciplinary rehabilitation; NC = nurse consultation; Δ score = change score; mean diff. = mean difference scores between MDR and NC; CIS-20R = Checklist Individual Strength; FSS = Fatigue Severity Scale; MFIS = Modified Fatigue Impact Scale; P = P value; + = positive change score; − = negative change score; For all variables higher scores indicate worse condition of the outcome assessed.

Table 3 shows the results for all other secondary outcomes. At 12–24 weeks, the DIP subscale ‘symptoms’ (P = 0.03), and the IPA problem experience subscale ‘mobility’ (P = 0.03) showed a significant difference in favour of the MDR group. No significant group differences were found for the other secondary measures of outcome.

**Table 3:** Other secondary outcomes at 12 and 24 weeks for people with MS randomized to multidisciplinary rehabilitation or Nurse consultation.

	MDR		NC		MDR		NC		Between-group differences	
	12 weeks(N = 21)	24 weeks(N = 21)	12 weeks(N = 23)	24 weeks(N = 23)	0–12 weeksΔ score	12–24 weeksΔ score	0–12 weeksΔ score	12–24 weeksΔ score	0–12 weeks Meandiff.; P	12–24 weeks Meandiff.; P
FIM	120 (7)	121 (6)	119 (9)	120 (11)	2 (4)	1 (4)	−1 (5)	−1 (9)	3.0; 0.13	2.0; 0.34
MSIS Physical	50 (22.5)	45 (19.5)	39 (28)	41 (21)	1 (7)	−3 (14)	2 (9)	1 (9)	3.0; 0.54	4.0; 0.50
MSIS Psychological	16 (8.75)	18 (5)	18 (11)	18 (12)	0 (6)	0 (6)	1 (5)	0 (7)	1.0; 0.33	0; 0.99
DIP Symptoms	0 (0.33)	0 (0.33)	0 (0.33)	0.33 (0.33)	−0.06 (0.20)	−0.05 (0.24)	0.01 (0.29)	0.12 (0.26)	0.07; 0.33	0.17; 0.03*
DIP Mobility	0 (0.15)	0 (0.10)	0 (0.10)	0.10 (0.10)	−0.01 (0.08)	−0.02 (0.09)	0.01 (0.08)	0 (0.12)	0.02; 0.40	0.02; 0.23
DIP Self-care	0 (0)	0 (0)	0 (0)	0 (0)	−0.02 (0.09)	−0.01 (0.07)	−0.01 (0.10)	0.01 (0.10)	0.01; 0.77	0.02; 0.50
DIP Social Activities	0 (0.1)	0 (0.10)	0 (0.10)	0 (0.10)	0.03 (0.18)	−0.04 (0.09)	0.03 (0.16)	−0.01 (0.13)	0; 0.81	0.03; 0.33
DIP Communication	0 (0)	0 (0.0)	0 (0)	0 (0)	0 (0)	0 (0)	0.02 (0.12)	−0.01 (0.07)	0.02; 0.18	0.01; 0.57
DIP Psychological status	0 (0.10)	0 (0.10)	0 (0)	0 (0)	0.07 (0.21)	−0.06 (0.23)	0.02 (0.12)	0.02 (0.16)	0.05; 0.64	0.08; 0.64
IPA subscales										
Autonomy indoors	0.29 (1.21)	0.71 (1)	0.86 (1.43)	1 (1)	0.01 (0.34)	0.12 (0.44)	−0.16 (0.46)	0.12 (0.50)	0.17; 0.19	0; 0.79
Family role	1.71 (1.28)	1.57 (1.07)	1.43 (1.43)	1.43 (1)	0.16 (0.79)	−0.10(0.76)	−0.05 (0.68)	−0.12 (0.61)	0.21,0.35	0.02; 0.25
Autonomy outdoors	1.8 (1.1)	1.8 (0.7)	1.40 (1)	1.40 (0.8)	0 (0.48)	0.07(0.60)	0.02 (0.58)	0.03 (0.58)	0.02; 0.84	0.04; 0.97
Social life & relationships	1.15 (0.71)	1.14 (0.36)	1 (0.86)	1 (0.86)	0.07(0.52)	0.01(0.45)	−0.08 (0.56)	0.04 (0.69)	0.15; 0.20	0.03; 0.62
Work & education	2 (0.93)	2.2 (1.46)	1.5 (1)	1.80 (1)	−0.6 (0.90)	0.16(0.58)	−0.30 (0.92)	0.31 (1.04)	0.3; 0.86	0.15; 0.79
IPA problem experience										
Mobility	1 (1)	1 (1)	1 (0)	1 (1)	0.1 (0.54)	−0.19 (0.60)	−0.13 (0.55)	0.22 (0.60)	0.23; 0.17	0.41; 0.03*
Self-care	1 (2)	1 (2)	1 (2)	1 (2)	0 (0.63)	0 (0.55)	0.17 (0.78)	−0.04 (0.98)	0.17; 0.57	0.04; 0.80
Activities around house	1 (1)	1 (0)	1 (0)	1 (1)	0.05 (0.67)	−0.14 (0.65)	0.09 (0.79)	0 (0.80)	0.04; 0.91	0.14; 0.46
Looking after money	1 (1)	1 (1)	1 (0)	1 (1)	0.1 (0.70)	0 (0.55)	−0.09 (0.67)	0.09 (0.79)	0.19; 0.27	0.09; 0.35
Leisure	1 (1)	1 (0)	1 (1)	1 (1)	0 (0.90)	−0.10 (0.54)	0.22 (0.60)	0.04 (0.71)	0.22; 0.32	0.14; 0.47
Social life & relationships	1 (1)	1 (2)	1 (1)	1 (1)	−0.05 (0.38)	−0.10 (0.70)	0 (0.74)	0.22 (0.80)	0.05; 0.81	0.32; 0.22
Helping, supporting others	1 (0.5)	1 (0)	1 (0)	1 (0)	−0.1 (0.44)	0 (0.55)	0.09 (0.51)	0 (0.80)	0.19; 0.21	0; 0.84
Paid or voluntary work	1 (2)	1 (1.5)	1 (2)	1 (1)	0.19 (0.81)	−0.05 (0.74)	0.09 (0.95)	−0.04 (0.82)	0.1; 0.96	0.01; 0.77
Education and training	1 (1)	1 (1)	0 (1)	0 (1)	−0.05 (0.92)	0.10 (0.83)	−0.13 (0.92)	−0.09 (0.79)	0.08; 0.94	0.19; 0.53

Values are medians (inter quartile ranges). Change scores are means (standard deviations). MDR = multidisciplinary rehabilitation; NC = nurse consultation; Δ score = change score; mean diff. = mean difference scores between MDR and NC; P = P value; FIM = functional independence measure; DIP = the Disability and Impact Profile, IPA = Impact on Participation and Autonomy;

* = significant at p<0.05;

+ = positive change score;

− = negative change score;

For all variables higher scores indicate worse condition of the outcome assessed.

No significant within-group effects were found for multidisciplinary rehabilitation or nurse consultation with respect to the primary (0.57≤p≤0.97) and secondary (0.11≤p≤0.92) outcome measures from baseline to 12 or 24 weeks.

## Discussion

The present study focussed on the effects of a multidisciplinary outpatient programme as compared to nurse consultation on chronic fatigue for people with MS. Our hypothesis that multidisciplinary rehabilitation would be more effective in terms of reducing self-reported fatigue in MS patients with chronic fatigue cannot be confirmed. Our results show that fatigue was quite invariant from baseline onwards, irrespective of the type of therapy applied. In fact, no differences were found not only on the primary outcome, the CIS-20R, but also on two other self-report fatigue questionnaires, the FSS and the MFIS. Since different fatigue questionnaires measure different aspects or constructs of fatigue [30], [40], selecting another primary outcome would have resulted in similar conclusions. In line with patients’ self-reported fatigue, no differences between the groups were found on the secondary outcomes, i.e. level of disability (FIM), impact of MS (DIP and MSIS), or patients’ perceived participation (IPA). Exceptions were the differential effects on the DIP subscale ‘symptoms’, and the IPA problem experience subscale ‘mobility’ at 24 weeks follow-up. These statistically significant differences may easily explained by chance alone due to multiple testing. Therefore, the two significant secondary results do not provide convincing evidence of any real treatment effects.

The reasons for this negative trial remain unclear, but are in line with those of Kos and colleagues [41], who also failed to find a difference in effects between a multidisciplinary fatigue management programme and a placebo intervention programme. However, our results contrast with theirs with regard to within group-effects. We also measured fatigue with the MFIS [31], [32] and found no change over time in either the MDR or the NC group. In the study by Kos et al [41], both groups showed similar statistically significant changes over time on the MFIS. Interestingly, Kos et al. [41] did not distinguish between acute or chronic fatigue, but patients were selected largely based on the severity of impact of fatigue over the previous month as assessed with the Guy’s Neurological Disability scale. An explanation of the different finding might be that our inclusion procedure was aimed at a more demarcated study sample of MS patients suffering from chronic fatigue, whose chronic fatigue was hardly influenced by our rehabilitation intervention. This in turn may support the idea that acute and chronic fatigue should be managed differently, with an initial focus on identifying and treating all factors that can contribute to the acute feelings of fatigue, and subsequently addressing the chronic aspects [3]. Furthermore, our results challenge the use of interventions such as aerobic training (AT) [14], [26], behavioural approaches or energy conservation management (ECM) [18], [19] to treat chronic fatigue. The quite invariant profile of chronic fatigue in the present trial, irrespective of the type of therapy applied, suggests the need for new interventions.

The present study had some limitations. The number of MS patients included in this trial was small. Despite the low numbers recruited, however, we believe that the results of our negative trial do show a lack of any trends favouring the effectiveness of our multidisciplinary rehabilitation approach when compared to nurse consultation. In addition, we recruited only ambulatory MS patients with chronic fatigue older than 18 years of age. This restriction limits the generalisation of the presents findings to non-ambulatory MS patients.

For patients allocated to MDR, the referral pathway by the multidisciplinary team was an integral part of the intervention. Although all members of the team are experts in the treatment of MS, we are unable to confirm that this procedure led to an optimal deployment of disciplines. The majority of participants in the multidisciplinary group received a comprehensive treatment combining different aspects of the fatigue treatment. This kind of comprehensive advice, partly based on different principles, however, did retrospectively show a large overlap with the MS nurse consultation. The operationalization of the current design may have contributed to a lack of contrast between multidisciplinary rehabilitation and nurse consultation.

Trials focussing on reducing fatigue in patients with MS [12], [17]–[19], [41] have included participants based on the impact of fatigue as measured with self-report scales like the MFIS and FSS. One may question if this inclusion method leads to a clear definition of chronic fatigue and factors secondary to MS that may contribute to feelings of fatigue. When it comes to including participants in trials, a combination of using descriptive modifiers and screening for factors contributing to fatigue may help to discriminate acute, intermittent fatigue from chronic persistent fatigue. Proper demarcation may in turn help distinguish between treatable and untreatable causes of fatigue in MS.

## Supporting Information

Protocol S1 **study protocol Efficacy of multidisciplinary treatment of fatigue in multiple sclerosis: a randomised controlled trial.**
(DOC)

Checklist S1 **CONSORT 2010 checklist of information to include when reporting a randomised trial.**
(DOC)

## References

[1] Chaudhuri A, Behan PO (2004) Fatigue in neurological disorders. Lancet 363 (9413): 978–988. 10.1016/S0140-6736(04)15794-2 15043967

[2] MacAllister WS, Krupp LB (2005) Multiple sclerosis-related fatigue. Phys Med Rehabil Clin N Am 16(2): 483–502. 15893683 10.1016/j.pmr.2005.01.014

[3] Vucic S, Burke D, Kiernan MC (2010) Fatigue in multiple sclerosis: mechanisms and management. Clin Neurophysiol 121(6): 809–817. 20100665 10.1016/j.clinph.2009.12.013

[4] Vercoulen JH, Hommes OR, Swanink CM, Jongen PJ, Fennis JF, et al (1996) The measurement of fatigue in patients with multiple sclerosis. A multidimensional comparison with patients with chronic fatigue syndrome and healthy subjects. Arch Neurol 53: 642–649. 8929171 10.1001/archneur.1996.00550070080014

[5] Flachenecker P, Kümpfel T, Kallmann B, Gottschalk M, Grauer O, et al (2002) Fatigue in multiple sclerosis: a comparison of different rating scales and correlation to clinical parameters. Multiple Sclerosis 8(6): 523–526. 12474995 10.1191/1352458502ms839oa

[6] Multiple Sclerosis Council for clinical practice guidelines (1998) Fatigue and multiple sclerosis: evidence-based management strategies for fatigue in Multiple sclerosis. Washington, DC: Paralyzed veterans of America.

[7] Kos D, Kerckhofs E, Nagels G, D’hooghe MB, Ilsbroukx S (2008) Origin of fatigue in multiple sclerosis: review of the literature. Neurorehabil Neural Repair 22(1): 91–100. 17409388 10.1177/1545968306298934

[8] Weinshenker BG, Penman M, Bass B, Ebers GC, Rice GPA (1992) A double blind, randomized, crossover trial of pemoline in fatigue associated with multiple sclerosis. Neurology 42: 1468–1471. 1641137 10.1212/wnl.42.8.1468

[9] Brañas P, Jordan R, Fry-Smith A, Burls A, Hyde C (2000) Treatments for fatigue in multiple sclerosis: a rapid and systematic review. Health Technol Assess. 4(27): 1–61. 11074395

[10] Rammohan KW, Rosenberg JH, Lynn DJ, Blumenfeld AM, Pollak CP, et al (2002) Efficacy and safety of modafinil (Provigil) for the treatment of fatigue in multiple sclerosis: a two centre phase 2 study. J Neurol Neurosurg Psychiatry 72: 179–183. 11796766 10.1136/jnnp.72.2.179 PMC1737733

[11] Stankoff B, Waubant E, Confavreux C, Edan G, Debouverie M, et al (2005) Modafinil for fatigue in MS: a randomized placebo-controlled double-blind study. Neurology; 12 64(7): 1139–1143. 10.1212/01.WNL.0000158272.27070.6A 15824337

[12] Van Kessel K, Moss-Morris R, Willoughby E, Chalder T, Johnson MH, et al (2008) A Randomized Controlled Trial of Cognitive Behavior Therapy for Multiple Sclerosis Fatigue. Psychosom Med 70: 205–213. 18256342 10.1097/PSY.0b013e3181643065

[13] Brown JN, Howard CA, Kemp DW (2010) Modafinil for the treatment of multiple sclerosis-related fatigue. Ann Pharmacother 44(6): 1098–103. 20442351 10.1345/aph.1M705

[14] Petajan JH, Gappmaier E, White AT, Spencer MK, Mino L (1996) Impact of aerobic training on fitness and quality of life in multiple sclerosis. Ann Neurol 39: 432–441. 8619521 10.1002/ana.410390405

[15] Oken BS, Kishiyama S, Zajdel D, Bourdette D, Carlesen J (2004) Randomized controlled trial of yoga and exercise in multiple sclerosis. Neurology 8 62(11): 2058–2064. 10.1212/01.wnl.0000129534.88602.5c 15184614

[16] Cakt BD, Nacir B, Genç H, Saraçoğlu M, Karagöz A (2010) Cycling progressive resistance training for people with multiple sclerosis: a randomized controlled study. Am J Phys Med Rehabil 89(6): 446–57. 20216060 10.1097/PHM.0b013e3181d3e71f

[17] Dalgas U, Stenager E, Jakobsen J, Petersen T, Hansen HJ (2010) Fatigue, mood and quality of life improve in MS patients after progressive resistance training. Mult Scler 16(4): 480–90. 20194584 10.1177/1352458509360040

[18] Mathiowetz VG, Finlayson ML, Matuska KM, Chen HY, Luo P (2005) Randomized controlled trial of an energy conservation course for persons with multiple sclerosis. Mult Scler 11(5): 592–601. 16193899 10.1191/1352458505ms1198oa

[19] Mathiowetz VG, Matuska KM, Finlayson ML, Luo P, Chen HY (2007) One-year follow-up to a randomized controlled trial of an energy conservation course for persons with multiple sclerosis. Int J Rehabil Res 30(4): 305–313. 17975450 10.1097/MRR.0b013e3282f14434

[20] Induruwa I, Constantinescu CS, Gran B (2012) Fatigue in multiple sclerosis - a brief review. J Neurol Sci 15 323(1–2): 9–15. 10.1016/j.jns.2012.08.007 22935407

[21] Neill J, Belan I, Ried K (2006) Effectiveness of non-pharmacological interventions for fatigue in adults with multiple sclerosis, rheumatoid arthritis, or systemic lupus erythematosus: a systematic review. J Adv Nurs 56(6): 617–35.Erratum in J Adv Nurs 2007 Jan 57(2): 225. 10.1111/j.1365-2648.2006.04054.x 17118041

[22] McDonald WI, Compston A, Edan G, Goodkin D, Hartung HP (2001) Recommended diagnostic criteria for multiple sclerosis: guidelines from the International panel on the diagnosis of multiple sclerosis. Ann Neurol 50: 121–127. 11456302 10.1002/ana.1032

[23] Zigmond AS, Snaith RP (1983) The Hospital Anxiety and Depression Scale. Acta psychiatr Scand 67: 361–370. 6880820 10.1111/j.1600-0447.1983.tb09716.x

[24] Savard J, Laberge B, Gauthier JG, Ivers H, Bergeron MG (1998) Evaluating anxiety and depression in HIV-infected patients. J Personality Assessm 7: 349–367. 10.1207/s15327752jpa7103_5 9933941

[25] Kubukeli ZN, Noakes TD, Dennis SC (2002) Training techniques to improve endurance exercise performances. Sports Med 32: 489–509. 12076176 10.2165/00007256-200232080-00002

[26] Mostert S, Kesselring J (2002) Effects of a short-term exercise training program on aerobic fitness, fatigue, health perception and activity level of subjects with multiple sclerosis. Mult Scler 8: 161–168. 11990874 10.1191/1352458502ms779oa

[27] Steultjens EM, Dekker J, Bouter LM, Cardol M, Van de Nes JC (2003) Occupational therapy for multiple sclerosis. Cochrane Database of Systematic Reviews CD003608. 10.1002/14651858.CD003608 PMC9022193 12917976

[28] McCloskey JC, Bulechek GM (2000) Nursing Interventions Classification (NIC) (3rd ed.). St. Louis: Mosby-Year Book. 8591448

[29] Vercoulen JH, Swanink CM, Fennis JF, Galama JM, van der Meer JW (1994) Dimensional assessment in chronic fatigue syndrome. J Psychosom Res 38: 383–392. 7965927 10.1016/0022-3999(94)90099-x

[30] Rietberg MB, Van Wegen EE, Kwakkel G (2010) Measuring fatigue in patients with multiple sclerosis: reproducibility, responsiveness and concurrent validity of three Dutch self-report questionnaires. Disabil Rehabil 32(22): 1870–1876. 20345240 10.3109/09638281003734458

[31] Kos D, Kerckhofs E, Nagels G, D'Hooghe BD, Duquet W (2003) Assessing fatigue in multiple sclerosis: Dutch modified fatigue impact scale. Acta Neurol Belg 103(4): 185–191. 15008502

[32] Kos D, Kerckhofs E, Carrea I, Verza R, Ramos M (2005) Evaluation of the Modified Fatigue Impact Scale in four different European countries. Mult Scler 11(1): 76–80. 15732270 10.1191/1352458505ms1117oa

[33] Krupp LB, LaRocca NG, Muir-Nash J, Steinberg AD (1989) The fatigue severity scale. Application to patients with multiple sclerosis and systemic lupus erythematosus. Arch Neurol 46(10): 1121–1123. 2803071 10.1001/archneur.1989.00520460115022

[34] Granger CV, Cotter AC, Hamilton BB, Fiedler RC, Hens MM (1990) Functional assessment scales: a study of persons with multiple sclerosis. Arch Phys Med Rehabil 71: 870–875. 2222154

[35] Lankhorst GJ, Jelles F, Smits RC, Polman CH, Kuik DJ, et al (1996) Quality of life in multiple sclerosis: the disability and impact profile (DIP). J. Neurology 243: 469–474. 10.1007/BF00900502 8803821

[36] Pfennings LE, Van der Ploeg HM, Cohen L, Bramsen I, Polman CH (1999) A health-related quality of life questionnaire for multiple sclerosis. Acta Neurol Scand 100(3): 148–155. 10478577 10.1111/j.1600-0404.1999.tb00730.x

[37] Hobart J, Lamping D, Fitzpatrick R, Riazi A, Thompson A (2001) The Multiple Sclerosis Impact Scale (MSIS-29): A new patient-based outcome measures. Brain 124, 962–973. 10.1093/brain/124.5.962 11335698

[38] Cardol M, Beelen A, Bos van den GAM (2001) The ability of the ‘Impact on Participation and Autonomy (IPA) questionnaire to detect improvement over time. In: Beyond disability: assessing participation and autonomy in medical rehabilitation. Thesis Department of Rehabilitation of the Academic Medical Centre in Amsterdam, Netherlands; 85–97.

[39] Werf SP van der, Jongen PJH, Lycklama A, Nijenholt GJ, Barkhof F (1998) Fatigue In multiple sclerosis: interrelations between fatigue complaints, cerebral MRI abnormalities and neurological disability. Journal of the Neurological Siences 160: 164–170. 10.1016/s0022-510x(98)00251-2 9849800

[40] Elbers RG, Rietberg MB, Van Wegen EE, Verhoef J, Kramer SF (2012) Self-report fatigue questionnaires in multiple sclerosis, Parkinson's disease and stroke: a systematic review of measurement properties. Qual Life Res 21(6): 925–44. 22012025 10.1007/s11136-011-0009-2 PMC3389599

[41] Kos D, Duportail M, D’hooghe M, Nagels G, Kerckhofs E (2007) Multidisciplinary fatigue management programme in multiple sclerosis: a randomized clinical trial. Mult Scler 13(8): 996–1003. 17623738 10.1177/1352458507078392

