# Supplementary material for: Effects of Multidisciplinary Rehabilitation on Chronic Fatigue in Multiple Sclerosis: A Randomized Controlled Trial
Source: PLoS One. 2014 Sep 18;9(9):e107710. doi: 10.1371/journal.pone.0107710 (PMC4169408; doi:10.1371/journal.pone.0107710)
Supplement: Protocol S1 — study protocol Efficacy of multidisciplinary treatment of fatigue in multiple sclerosis: a randomised controlled trial. (DOC) [file pone.0107710.s001.doc]

#

# PROTOCOL

**Efficacy of multidisciplinary treatment of fatigue in multiple sclerosis:**

**a randomised controlled trial.**

Project leader: G. Kwakkel, PhD

##### Research coordinator Department of physical therapy

##### VU University Medical Centre

De Boelelaan 1117, 1081 HV Amsterdam

The Netherlands

direct tel no: +31-20-4440460

direct fax no: +31-20-4440460

E-mail: [g.kwakkel@vumc.nl](mailto:g.kwakkel@vumc.nl)

**Summary**

In an effort to optimise the management of fatigue in multiple sclerosis (MS), a multidisciplinary treatment programme has been developed in the MS Centre of the VU university medical center (VUmc). This treatment programme comprises a standardised fatigue questionnaire, a referral schedule to paramedical disciplines and the following treatment by these disciplines. Factors that may contribute to MS related fatigue are identified and the patient is then referred to the disciplines that are professionally linked to these factors. The objective of the present study is to investigate the efficacy of the outpatient multidisciplinary treatment by the paramedical disciplines on chronic fatigue in MS. For this purpose, the multidisciplinary treatment (i.e., experimental intervention) will be compared with a consultation programme offered by an MS-nurse as control condition. At least 48 MS patients with fatigue will be randomly assigned to a four months period of multidisciplinary treatment or to the control treatment. As primary outcome, fatigue will be measured with the Checklist Individual Strength (CIS-20r). Secondary outcomes will be assessed by the Modified Fatigue Impact Scale (MFIS), the Fatigue Severity Scale (FSS), the Functional Independence Measure (FIM), the Disability and Impact Profile (DIP), the Medical Outcome 36-item study Short-Form Health Survey Questionnaire (SF-36) and the Impact on Participation and Autonomy (IPA), respectively. Demographic variables, disease characteristics and the EDSS of the participating subjects will be compared between the experimental and control groups to verify prognostic comparability at the baseline. Differences in change scores between the two interventions will be compared at baseline, 3 and 6 months.

**Introduction**

Multiple sclerosis (MS) is a chronic neurological disease, which usually affects relatively young adult individuals. In the Netherlands, approximately 161 out of 100,000 people suffer from MS [1]. Fatigue is a common complaint in MS patients, reported by 76 to 92% of the patients [2], whereas between 55 and 75% of them consider fatigue as one of the most debilitating symptoms [2]. The cause of fatigue in MS is not entirely understood. While considered to be inherent to MS, fatigue can also be affected by factors secondary to MS, such as physical (e.g. deteriorating condition), psychological (e.g. stress, depression) and personal factors (e.g. career, relatives). No relation was found between fatigue and degree of disability or degree of abnormality on magnetic resonance imaging [3].

There is no simple treatment for MS-related fatigue. Although several drugs such as amantadine have been advocated with respect to their effect on fatigue in MS, fatigue responds to pharmacological treatment in only a small percentage of patients [4, 5, 6]. Recently, the impact of nursing interventions in fatigue have been put down in the Nursing Intervention Classification (NIC) [7] and consist of consultation with respect to energy conservation, exercise, mood management, nutrition and sleep. While commonly used in clinical practice, the efficacy of nursing interventions has not been investigated. Other non-pharmacological treatments like aerobic training [8] and energy management strategies [9] were found to be effective but have not been investigated in randomised controlled trials. The effect of psychotherapeutic intervention on fatigue has not been studied in MS specifically. In chronic fatigue syndrome however, the effect of cognitive behaviour therapy has produced positive effects [10].

Considering the multidimensional character of fatigue in MS, it seems obvious to treat fatigue in a tailored, multidisciplinary way. To date, no research has been conducted on the effects of a multidisciplinary treatment programme for fatigue in MS.

The objective of the present study is to investigate the efficacy of multidisciplinary treatment of chronic fatigue in MS, compared to consultation by an MS nurse. A schematic representation of the study procedure can be found in appendix 1. The structured multidisciplinary treatment that will be studied has been developed by the VUmc MS Centre and consists of a fatigue questionnaire to identify factors contributing to fatigue and a referral schedule to the appropriate discipline(s) (Appendix 2).

After systematically screening patients on symptoms of fatigue and subsequent referral, the participating disciplines will treat MS-related fatigue according to specific treatment programmes (Appendix 3). The present study will focus on investigating the efficacy of the package of treatment programmes of the participating disciplines.

The control treatment will be applied by the MS-nurse (Appendix 4) and consists of regular consultation. These consultations will contain the guidelines as proposed by the NIC [7]

**METHODS**

Patients

Patients diagnosed with MS according to the criteria of Poser [12] or McDonald [13] and suffering from chronic fatigue will be included in the present trial. According to the Multiple Sclerosis Council for Clinical Practice Guidelines [11] chronic fatigue will be defined as “*a significant problem present for any amount of time on 50 percent of the days, for more than 6 weeks*”. There are no restrictions for sex or type of MS. The patients will be recruited from the outpatient clinic of the VU University Medical Centre in Amsterdam

The exclusion criteria are:

- No informed consent to participate in the trial
- Acute fatigue (defined as: new or a significant increase in feelings of fatigue in the previous 6 weeks [11]).
- Current MS relapse
- Current pregnancy
- Current alcohol or substance abuse
- Other physical conditions importantly contributing to fatigue, like spasm
- Depressive mood disorder
- Age under 18 years

### Intervention

Patients will be screened on symptoms of fatigue (Appendix 2). The multidisciplinary team discusses the results of the screening by the neurologist and the pathway of referral will be determined for each patient individually. Patients will be referred to occupational therapy or to physical therapy or to social work or to a combination of these treatments. The participating disciplines will treat MS-related fatigue according to specific treatment programmes (Appendix 3). Information concerning treatment frequencies and time are kept in a diary. The diary will also be used for any changes in medication.

**Control treatment**

MS-nurse consultation, with respect to energy conservation, exercise, mood management, nutrition and sleep (see Appendix 4). Information concerning treatment frequencies and time are kept in a diary.

### Outcomes

### Baseline demographic and disease characteristics:

### Sex, age, clinical type of MS, years since first symptom, years since diagnosis, medication and estimated score on the EDSS [15] will be documented.

Primary measurement of outcome:

- Checklist Individual Strength (CIS-20r) [16] is a multidimensional instrument for the measurement of fatigue. It consists of 20 items, which are scored on a seven-point scale. It has been validated for MS patients in a Dutch version, it is easy to complete and has good psychometric properties. As it has not been validated in other languages, its use in international literature is limited.

Secondary measurements of outcome:

- Modified Fatigue Impact Scale [33, 34] is shortened version (21-items) of the 40-item Fatigue Impact Scale [17]. In 1998 the panel of the MS Council for Clinical Practice Guidelines recommend the MFIS for clinical use and for research. Like the FIS the MFIS evaluates the impact of fatigue on physical, cognitive an psychosocial function. Every item is scored on a five-point Likert scale (range 0-4). The Dutch version of the MFIS is a reliable, valid and responsive tool to assess the impact of MS-related fatigue on daily life [34]
- Fatigue Severity Scale (FSS) [35] is a nine-item questionnaire to assess the severity of fatigue and its impact on an individual’s daily functioning using a seven-point rating scale. The FSS has become one of the most commonly used measurements of fatigue severity in MS patients [35]. It was designed for MS patients and patients with chronic fatigue syndrome [18]. The FSS has been shown to have a high degree of validity and sensitivity to clinical changes. A Dutch version has recently been validated [personal communication].

- Functional Independence Measure (FIM) [19] measures activities of daily living. It is an 18-item, generic seven-point rating scale to assess physical and cognitive disability in terms of burden of care. The FIM has been applied to MS patients [19] and its reliability [20] and validity [20, 21] are established.

- The Disability and Impact Profile (DIP) [22] is a 39 item self-administered questionnaire regarding activities that may be restricted by a disabling disease. Each item is rated on a 0-10 point scale for its current disability and for the importance of that disability. The DIP will be enlarged with two questions as proposed by Pfennings in her study on Health-related quality of life in patients with multiple sclerosis [23]. The validity of the DIP is satisfactory [24, 25] and the reliability acceptable [26].

- The Medical Outcome 36-item study Short-Form Health Survey Questionnaire (SF-36) [27] will be used to measure health-related quality of life. The self-administered SF-36 rates general health perception. It consists of eight multi-item subclasses in two dimensions, physical and mental. Each dimension item score is coded, added and transformed to a 0-100 scale. The SF-36 is a reliable and valid measure to determine self-perception of health status. It has been widely applied in MS patients. Part of the SF-36 was considered to be an important measure of quality of life in MS [23].
- The Impact on Participation and Autonomy (IPA) measures person-perceived participation [28]. It is a self-administered generic questionnaire, addressing the impact of a condition on two different aspects of participation. One aspect is perceived participation and autonomy, measured with 31 items and scored on a five-point rating scale. The other aspect, related problem-experience for aspects of participation, is measured for eight problems on a three-point rating scale. The IPA is able to detect important within-person improvement over time and its reliability and validity are good [28, 29].

## Design

A randomised controlled trial with repeated measures (figure 1) will be used to address the research question. The trial protocol requires approval from the local research ethics committee of the VU University Medical Centre.

Pre-randomisation

Patients with chronic fatigue will be screened by the neurologist, in order to check the inclusion and exclusion criteria for participation in the present study. Screening for depressive symptoms is assessed with the Hospital Anxiety and Depression Scale (HADS) by the independent observer. The HADS is a 14-item self-report scale designed to measure depressive symptoms in a hospital setting [14]. A score of 8 or higher on the HADS suggests a depression and will exclude the patient from the current study.

Randomisation

Patients will be randomised to the multidisciplinary treatment (MDT) or control treatment (CT) group. The randomisation procedure is based on random number tables and will be performed by sealed, opaque envelopes.

After randomisation

After finishing baseline measurements and the randomisation procedure (Figure 1), the neurologist will complete the standardised fatigue questionnaire for the MDT group. The multidisciplinary team discusses the results of the screening by the neurologist and the pathway of referral will be determined for each patient individually. Subsequently, an independent observer will achieve patients’ informed consent for participation in the trial and measure the CIS-20r, MFIS, FSS, FIM, DIP, SF-36, IPA and EDDS-score. The baseline assessment will be twice: at inclusion and precisely at the day of the start of the treatment (proximately 1 week) These two baseline assessment are done to correct for possible impact of increased attention at the pre-treatment stage.

The amount and frequencies of therapy in the MDT group will be documented in a patient’s diary. A MS-nurse will see the CT group on a monthly basis. The outcomes will be evaluated by an independent observer at baseline, at 3 and at 6 months after randomisation. The observer will be blinded for treatment assignment. The assessment are at 3 months, because some effect can be expected at that time. Assessment at 6 months is done to assess if the effects seen at 3 months are lasting.

The assessments will be combined as much as possible with regular visits at the VU University Medical Centre; if this is not possible the patient will be offered payment of travel expenses.

**Figure 1:** Schematic representation of the current trial. R = randomisation, M = assessment moment, MDT = Multidisciplinary treatment, CT = control treatment.

Power analyse

A minimum of 48 patients (including 10% dropout) will be recruited into the trial. This number is required in order to get sufficient power (minimum 0.80 (1-β) power value) in detecting differences in efficacy between the two treatment conditions with respect to a 10% difference on the primary measurement of outcome CIS-20r. Analysis will be based upon an alpha of 0.05 and assuming to find a medium sized effect (defined as: difference between group means, divided by the average population variance at the baseline).

To reach a total of 48 patients, the estimated inclusion period is 2.5 years.

## Statistical analysis

The statistical analysis will be carried out by using the SPSS computer programme, version 11. Demographic and disease characteristics of the subjects at baseline will be compared between the treatment group (MDT) and the control group (CT) by Mann-Whitney U test for ordinal variables and by Χ^2^ test for categorical variables.

Outcome scores will be analysed in terms of difference in scores between baseline, month 3 and month 6, respectively, by comparing differences between MDT and CT with respect to scores from the primary measurement of outcome the CIS-20r and the secondary measurements of outcomes MFIS, FSS, FIM, DIP, SF-36 and IPA. Possible differences in change scores will be tested two tailed with the Mann-Whitney U tests for their significance.

**Justification**

Personnel

Measurements will be done by an independent observer, who will be trained to assess the measures used in the study. Analysis and writing will be done by the project group. Since the interventions are part of the regular treatment, time burden of the study for the participating disciplines will be minimal.

Time frame

January 2005 – June 2005: preparations for the study

July 2005 – December 2008: inclusion, data collection, data entry

January 2009 – March 2009: data analysis and publication

**References**

[1] Minderhoud JM, Zwanikken CP. Volksgezondheid Toekomst Verkenning. *Nationaal kompas volksgezondheid.* Bilthoven: RIV. [www.nationaalkompas.nl](http://www.nationaalkompas.nl/)

[2] Krupp LB, Alvarez LA, LaRocca NG, Scheinberg LC. Fatigue in multiple sclerosis. *Arch Neurol* 1988; 45: 435-437.

[3] Werf van der SP, Jongen PJ, Lyckama A, Nijeholt GJ, Barkhof F, Hommes OR, Blijenberg G. Fatigue in multiple sclerosis: interrelations between fatigue complaints, cerebral MRI abnormalities and neurological disability. *J Neurol Sci* 1998; 8: 164-170.

[4] Rammohan KW, Rosenberg JH, Lynn DJ, Blumenfeld AM, Pollak CP, Nagaraja HN. Efficacy and safety of modafinil (Provigil) for the treatment of fatigue in multiple sclerosis: a two centre phase 2 study. *J Neurol Neurosurg Psychiatry* 2002; 72: 179–83.

[5] Weinshenker BG, Penman M, Bass B, Ebers GC, Rice GPA. A double blind, randomised, crossover trial of pemoline in fatigue associated with multiple sclerosis. *Neurology* 1992; 42: 1468–71.

[6] Canadian MS Research Group. A randomised controlled trial of amantadine in fatigue associated with MS. *Can J Neurosci* 1987; 14: 273–78.

[7] McCloskey & Bulechek (eds): Nursing Intervention Classification: the Iowa Intervention project. Mosbey 2000; St Louis, USA.

[8] Petajan, JH, Gappmaier, E, White, AT, Spencer, MK, Mino, L, Hicks, RW. Impact of aerobic training on fitness and quality of life in multiple sclerosis: *Ann Neurol* 1996; 39: 432-441.

[9] Mathiowetz V, Matuska KM, Murphy ME. Efficacy of an energy conservation course for persons with multiple sclerosis. *Arch Phys Med Rehabil* 2001; 82(4): 449-56.

[10] Prins J, Bleijenberg G, Bazelmans E, et al. Cognitive behaviour therapy for chronic fatigue syndrome: a multicentre randomised controlled trial. *Lancet* 2001; 357: 841-847.

[11] Fatigue and multiple sclerosis. Evidence-based management strategies for fatigue in Multiple sclerosis. *Multiple Sclerosis Council for clinical practice guidelines*, 1998.

[12] Poser C, Paty D, Scheinberg L, et al. New diagnostic criteria for multiple sclerosis: guidelines for research protocols. *Ann Neurol* 1983; 13: 227-231.

[13] McDonald WI, Compston A, Edan G, et al. Recommended diagnostic criteria for multiple sclerosis: guidelines from the International panel on the diagnosis of multiple sclerosis. *Ann Neurol* 2001; 50: 121-127.

[14] Savard J, Laberge B, Gauthier JG, et al. Evaluating anxiety and depression in HIV-infected patients. *J Personality Assessm* 1998; 7: 349-367.

[15] Kurtzke J. Rating neurologic impairment in multiple sclerosis: an expanded disability status scale (EDSS). *Neurology* 1983; 33: 1444-1452.

[16] Vercoulen JH, Swanink CM, Galama JM, Fennis JF, van der Meer JW, Bleijenberg G. Dimensional assessment in chronic fatigue syndrome. *J Psychosom Res* 1994; 38: 383-392.

[17] Fisk JD, Ritvo PG, Ross L, et al. Measuring the functional impact of fatigue: initial validation of the fatigue impact scale. *Clin Infect Dis* 1994; 18 suppl 1: S79-83.

[18] Schwid SR, Covington M, Segal BM, Goodman AD. Fatigue in Multiple Sclerosis: Current understandings and future directions. *J Rehab Res Devel* 2002; 29(2): 211-224.

[19] Granger CV, Cotter AC, Hamilton BB, Fiedler RC, Hens MM. Functional assessment scales: a study of persons with multiple sclerosis. *Arch Phys Med Rehabil* 1990; 71: 870-875.

[20] Hamilton BB, Laughlin JA, Granger CV, et al. Interrater agreement of the seven level Functional Independence Measure (FIM) *Arch Phys Med Rehabil* 1991; 72:790.

[21] Dodds TA, Martin DP, Stolov WC, et al. A validation of the Functional Independence Measurement and its performance among rehabilitation inpatients. *Arch Phys Med Rehabil* 1993; 74: 531-536.

[22] Lankhorst GJ, Jelles F et al. Quality of life in multiple sclerosis: the disability and impact profile (DIP). J. Neurology 1996; 243: 469-474.

[23] Pfennings LE, Van der Ploeg HM, Cohen L, Bramsen I, Polman CH, Lankhorst GJ, Vleugels L. A health-related quality of life questionnaire for multiple sclerosis. *Acta Neurol Scand* 1999; 100(3): 148-155.

[24] Cohen L, Pouwer F, Pfennings LE, Lankhorst GJ, Van der Ploeg HM, Polman CH et al. Factor structure of the Disability and Impact Profile in patients with multiple sclerosis. *Qual Life Res* 1999; 8(1-2):141-150.

[25] Lankhorst GJ, Jelles F, Smits RC, Polman CH, Kuik DJ, Pfennings LE et al. Quality of life in multiple sclerosis: the disability and impact profile (DIP). *J Neurol* 1996; 243(6):469-474.

[26] Pfennings L, Cohen L, van der PH, Polman C, Lankhorst G. Reliability of two measures of health-related quality of life in patients with multiple sclerosis. *Percept Mot Skills* 1998; 87(1):111-114.

[27] Ware JE, Snow KK, Kosinski M, Gandek B. SF-36 Health Survey: manual and interpretation guide. Boston: The Health Institute, New England Medical Centre, 1993.

[28] Cardol M, Beelen A, Bos van den GAM, Jong de BA, Groot de IJM, Haan de RJ. The ability of the ‘Impact on Participation and Autonomy (IPA) questionnaire to detect improvement over time. In: *Beyond disability: assessing participation and autonomy in medical rehabilitation*. Thesis Department of Rehabilitation of the Academic Medical Centre in Amsterdam, Netherlands 2001; 85-97.

[29] Cardol M, Haan de RJ, Jong de BA, Bos van den GAM, Groot de IJM. Psychometric properties of the ‘Impact on Participation and Autonomy (IPA) questionnaire. *Arch Phys Med Rehabil* 2001; 82: 210-216.

[30] Kubukeli ZN, Noakes TD, Dennis SC. Training techniques to improve endurance exercise performances. *Sports Med* 2002; 32:489-509.

[31] Mostert S, Kesselring J. Effects of a short-term exercise training program on aerobic fitness, fatigue, health perception and activity level of subjects with multiple sclerosis. *Mult Scler* 2002; 8:161-8.

[32] Mulcare JA. Multiple Sclerosis. In: American College of Sports Medicine. ACSM's exercise management for persons with chronic disease and disabilities. Champaign: Human Kinetics. 2^nd^ Edition, 2003.

[33] Kos D, Kerckhofs E, Carrea I, Verza R, Ramos M, Jansa J. Evaluation of the Modified Fatigue Impact Scale in four different European countries. Mult Scler. 2005 Feb;11(1):76-80.

[34] Kos D, Kerckhofs E, Nagels G, D'Hooghe BD, Duquet W, Duportail M, Ketelaer P. Assessing fatigue in multiple sclerosis: Dutch modified fatigue impact scale. Acta Neurol Belg. 2003 Dec;103(4):185-91.

[35] Krupp LB, LaRocca NG, Muir-Nash J, Steinberg AD. The fatigue severity scale. Application to patients with multiple sclerosis and systemic lupus erythematosus. *Arch Neurol* 1989; 46: 1121-1123.

Appendix 1: Multidisciplinary referral schedule for MS related chronic fatigue.

HADS

- HADS ≥ 8

**Psychologist**

Diagnostics & Treatment

**Neurologist:**

Exclude somatic factors

- Pain
- Spasticity
- Infection
- Exacerbation

**Neurologist & MS nurse:**

Fatigue is perceived by the patient as a major problem

**Neurologist / Physiatrist**:

Diagnostics & Treatment

S

T

U

D

Y

***I***

***N***

***T***

***E***

***R***

***V***

***E***

***N***

***T***

***I***

***O***

***N***

***C***

***O***

***N***

***T***

# *R*

***O***

***L***

*Presence of disabling physical functioning*

- *Lack of condition*

*Presence of disabilities in daily activities*

- *Inadequate balance rest/activity*
- *Inadequate energy use, & management*
- *Problems in self-care, productivity, leisure, mobility*

*Presence of disabling social functioning*

- *Insufficient support in social environment*
- *Stressful contact with services*
- *Coping habituation*

***Occupational therapist***

*Fatigue management*

- *Energy conservation*
- *Time management*
- *Efficient body mechanics*
- *Adaptation of equipment & environment*

***Physical therapist***

- *Reconditioning*

***Social worker***

- *Coping skills*
- *Coaching and coordinating social support*

No

**Neurologist*:***

Fatigue questionnaire

**MDTeam**

referral plan

Yes

Yes

Yes

Randomisation

***MS-nurse***

*Interventions concerning: Disturbed Rest / sleep-food imbalance, stimulating substances (abuse), general principles of medication management, pacing, priority setting, physical activity*

Appendix 2: Fatigue questionnaire and referral schedule

1 What factors do you consider of influence on your fatigue?

- - ...............
  - ...............
  - ...............

2 Are you capable of making a good division between rest and activity. For example, do you make priorities and do you set bounds?

- Yes
- No (referral to occupational therapist)

3 Does fatigue influence your performance of work or small jobs (paid or unpaid, at home or out of doors), your education, your leisure time or your social conduct?

- - Yes (referral to occupational therapist)
  - No

4 Does fatigue influence your sitting, standing or moving in or out of doors?

- Yes (referral to occupational therapist)
- No

5 Does fatigue influence your self-care, like bathing, dressing or facial care?

- Yes (referral to occupational therapist)
- No

6 Are you tired quickly by physical exercise or physical activity, i.e. by activities that result into deepened breathing and quicker heart beating?

- Yes
- No (continue with question 7)

6a Do you consider your physical condition adequate?

- - Yes
  - No (referral to physical therapist)

6b Do you think you could exercise more than you do now, without influencing your daily schedule too much?

- - Yes (continue with question 7)
  - No

6c Do you perform physical activity, which strains you modestly, for more than half an hour each day?

- - Yes
  - No (referral to physical therapist)

##### 7 Do you believe that you get insufficient support from your environment?

- - Yes
  - No (continue with question 8)

7a Do you feel that the lack of support influences your feelings of fatigue?

- - - Yes (referral to social worker)
    - No

8 Do you have, due to MS, long lasting (during more than 6 months) problems or conflicts at work or with social services (municipal authority, social security, etc)?

- - Yes
  - No (continue with question 9)

8a Do you feel that these problems at work reinforce your feelings of fatigue?

- Yes (referral to social worker)
- No

9 Do you feel that you have difficulties coping with MS?

- - Yes
  - No (continue with questionnaire summary)

9a Do you feel that this coping problem reinforces your feelings of fatigue?

- - - Yes (referral to social worker)
    - No

9b Do you wake up at night because of worrying for the future?

- Yes (referral to social worker)
- No

Continue with the summary of the fatigue questionnaire.

**Summary of fatigue questionnaire and referral schedule**

- Complete the points of attention according to the questionnaire.
- Ask the patient: Which points of attention do you consider the most important ? (indicate their sequence if possible)
- Ask the multidisciplinary team: which points of attention deserve priority?

| **Subject** | **Point of attention according to questionnaire** | | **Sequence patient** | **Sequence multi-disciplinary team** | **Referral discipline** |
| --- | --- | --- | --- | --- | --- |
|  | **Yes** | **No** |  |  |  |
| Division rest/activity | ◘ | ◘ |  |  | Occupational therapy  ◘ yes  ◘ no  ◘ ........... |
| Work, small jobs, education, leisure time, social contacts | ◘ | ◘ |  |  |  |
| Sitting and walking in- and out of doors | ◘ | ◘ |  |  |  |
| Personal care | ◘ | ◘ |  |  |  |
| Physical exercise / condition | ◘ | ◘ |  |  | Physical therapy  ◘ yes  ◘ no  ◘ ........... |
| Support from environment | ◘ | ◘ |  |  | Social work  ◘ yes  ◘ no  ◘ ........... |
| Conflicts/problems at work or with social services | ◘ | ◘ |  |  |  |
| Coping with MS | ◘ | ◘ |  |  |  |

**Appendix 3: Summary of treatment of fatigue by the individual disciplines participating in the multidisciplinary team**

# Physical therapy

For the indication ‘reconditioning’, an individual exercise training programme is constructed in order to improve physical fitness. Exercise training consists of twice-weekly supervised aerobic training sessions in circuit style performed individual or in classes. The training will consist of two sessions of 45 minutes per week, with a total length of 12 weeks. Various fitness devices (e.g. bicycle ergometer, row ergometer, stair walker, etc) will be used in blocks of six minutes, in order to offer a total body work-out and to train large muscle groups of the arms, legs and trunk. The maximal aerobic capacity of each participant will be tested to individually guide the training intensity. Moderate intensity is defined as 50-70% VO2peak steady state endurance training. This form of training has been shown to improve VO2peak and the Ventilatory Threshold [30, 31], its intensity is low enough to be tolerated by MS patients who suffer from chronic fatigue [8, 31] and it is recommended for improving aerobic capacity in MS patients [32].

Occupational therapy

Fatigue can have impact on the ability to choose, organise and perform activities. This can throw up many practical problems, for example in self-care, mobility, work, household and in socialisation and leisure activities. The occupational therapist can offer the patient a range of interventions to cope with the impact of fatigue and to perform activities that are important to the patient and his family. Fatigue management skills are taught to help with the application of coping strategies, energy conservation, time management, efficient body mechanics and task performance. Patients learn to use a range of self-care behaviours and work simplification principles like learning to recognise personal limits, scheduling activities, organising home and work, undertaking prolonged tasks in steps, efficient performance and using compensatory techniques and assistive devices to conserve energy.

**Social work**

Social workers are skilled in providing psychosocial support through counselling and practical assistance. Practical and financial problems, for example, being unable to work or having conflicts at work, problems with social services or having normal family life interrupted, can place enormous strain on families. Social workers can offer a model of intervention that addresses both emotional issues and practical concerns. Emotional support in the form of skilled listening, encouragement to ventilate, normalisation of feelings and advice on coping strategies, coupled with practical help will do much to enable both patient and family to cope with difficult circumstances.

**Appendix 4: Summary of treatment of fatigue by the MS nurse**

**MS nurse**

Nursing interventions for the patient with fatigue are based on general teaching principles of pacing, priority setting, and energy conservation and medication management. Pacing involves advising the patient in planning their activities for the day, allowing short rest periods (naps) so that fatigue produced by over activity is prevented. Priority setting is when advice is given in assisting patients to priorities tasks so that daily activities can be more easily accomplished and to plan high-priority tasks when their energy level is at its peak.

Energy conservation includes advice regarding the acceptance of help with daily life activities (this can be provided for example by their partner, significant others or healthcare professionals). Physical activity including sports is recommended as very essential. Activities resulting in a positive mood change such as listening to music, reading and yoga are openly discussed. General advice is given to improve their home and work environment (to save energy by using for example a dishwasher, and to prevent body heat by using air-condition/ventilation). Patients are taught to eat healthy, nutritious meals and to avoid weight gain or loss, and to be careful with alcohol and drug intake (abuse). If there is an insomnia problem or disrupted sleep pattern, the patient is taught sleeping habits (like to avoid caffeine use and to distress before sleeping) and in general recommended to have a regular sleep and daily routine. Medication management is given by advising how to take (MS) medication according to their individual needs.

All items mentioned above will be discussed with the patient during the first consultation. Monthly consultation will take place.

**Participants**

| Name | field of work | funded by institute | hrs/wk |
| --- | --- | --- | --- |
| *Project group:*   - Eyssen ICJM. Drs. - Jelles B. Dr. - Kwakkel G. Dr. - Rietberg MB. Drs. | Occupational therapy  Neurology  Research co-ordinator  Physiotherapy | VU University Medical Centre | 1  1  1  1 |
|  |  |  |  |
| *Support group:*   - Bakker-Latour KAA. - Bakker-Renema S. - Collete EH. Dr. - Egmond-van Zanten EA. - Groot de V. Drs. - Heeres I. - Hoogakker RG. - Kaufman HJ. Drs. - Klaveren van R. - Lankhorst GJ.Prof. Dr. - Ploeg van der HM. Prof. Dr - Polman CH. Prof. Dr. | Nursing  Occupational therapy  Psychology  Neurology  Rehabilitation medicine  Nursing  Social work  Social work  Physiotherapy  Rehabilitation medicine  Psychology  Neurology | VU University Medical Centre | 2  Advising  Advising  Advising  Advising  2  1  Advising  Advising  Advising  Advising  Advising |
